# Neonatal Hyperoxic Exposure Persistently Alters Lung Secretoglobins and Annexin A1

**DOI:** 10.1155/2013/408485

**Published:** 2013-09-26

**Authors:** Thomas M. Raffay, Morgan L. Locy, Cynthia L. Hill, Nik S. Jindal, Lynette K. Rogers, Stephen E. Welty, Trent E. Tipple

**Affiliations:** ^1^Nationwide Children's Hospital, The Ohio State University College of Medicine, Columbus, OH 43205, USA; ^2^The Research Institute at Nationwide Children's Hospital, 575 Children's Crossroad, Columbus, OH 43215, USA; ^3^Baylor College of Medicine, Houston, TX 77030, USA

## Abstract

Altered functions of the lung epithelial surface likely contribute to the respiratory morbidities in infants with bronchopulmonary dysplasia (BPD). Infants with BPD exhibit decreased expressions of secretoglobins (SCGBs), including Clara cell secretory protein (CCSP). Expression of lung SCGB and annexin A1 (ANXA1) is persistently altered in CCSP knockout mice suggesting that CCSP indirectly influences innate immune responses. The present studies tested the hypothesis that neonatal hyperoxic exposure induces deficits in CCSP expression that are associated with persistent alterations in lung SCGB and ANXA1 expression. Newborn C3H/HeN mice were exposed to room air (RA) or 85% O_2_ from birth and were sacrificed at 14 d or returned to RA for 14 d. Neonatal hyperoxia followed by RA recovery was associated with decreased lung CCSP and SCGB3A1 protein but not mRNA expression. Hyperoxia-induced alterations in the charge characteristics of ANXA1 were unchanged by RA recovery and were associated with elevated lung macrophage numbers. These findings support a model in which hyperoxia-induced alterations in Clara cell function influence lung innate immune function through effects on immunomodulatory proteins. Studies to determine the mechanism(s) by which CCSP alterations affect SCGBs, ANXA1, and innate immune responses in BPD are warranted.

## 1. Introduction

Bronchopulmonary dysplasia (BPD) affects approximately 14,000 preterm infants in the United States annually and is characterized by impaired alveolar and vascular development resulting in alveolar simplification with enlarged distal airspaces [1]. Infants with BPD are at increased risk of hospital readmission that is largely attributable to increases in the incidence and severity of respiratory infections [2–4]. As the first line of defense, proper immune function of the pulmonary epithelium, including coordinated secretion of cytokines, chemokines, and immunomodulatory proteins, is necessary for effective clearance of respiratory pathogens. Alterations in the homeostatic functions of the epithelial surface via direct injury or dysregulation of immune responses likely contribute to the enhanced respiratory morbidities experienced by infants with BPD, especially those imposed by lower tract viral disease.

Clara cells, nonciliated secretory cells in the conducting airways and trachea, protect the lung by producing proteins which contribute to mucosal defense through effects on immune responses [5]. Most properties of Clara cells are attributed to their primary protein products, the secretoglobins (SCGB). The best-characterized member of the SCGB family, SCGB1A1, is also called Clara cell secretory protein (CCSP). In the first week of life, findings of an inverse correlation between tracheal aspirate CCSP protein levels and O_2_ requirement in premature infants [6] support a role for CCSP deficiency in the pathogenesis of BPD. Furthermore, tracheal aspirate and cord blood CCSP expressions have been reported to predict the risk of developing BPD [7, 8]. Adult CCSP knockout (−/−) mice exposed to 100% O_2_ display increased mortality which is preceded by earlier onset of both cytokine expression and lung edema when compared to wild-type controls [9].

In addition to CCSP, other members of the SCGB protein family, specifically SCGB3A1 and SCGB3A2, are produced by distinct populations of Clara cells and are proposed to regulate local immune responses within the lung [10]. Lungs collected at autopsy from human infants with BPD exhibit quantitative differences in CCSP, SCGB3A1, and SCGB3A2 expressions in the surface epithelium of the upper and lower airways when compared to specimens obtained from unaffected infants [11]. CCSP−/− mice display enhanced inflammatory responses, increased susceptibility to oxidants, and decreased resistance to infection by microorganisms [12].

The significance of proper CCSP expression in response to pulmonary infection is suggested by increases in inflammatory responses to intratracheal adenovirus [13] and a more pathogenic response to respiratory syncytial virus (RSV) infection [11] in CCSP−/− mice than in wild-type mice. Recent studies suggest that CCSP deficiency indirectly influences the function of other epithelial and inflammatory cells through paracrine effects resulting in alterations in the protein products of the affected cells [14]. The absence of CCSP in CCSP−/− mouse lungs is associated with increased SCGB3A1 and SCGB3A2 mRNA levels [12]. In addition, loss of Clara cell secretory function alters macrophage function, exacerbates inflammatory responses, [15] and is directly related to posttranslational modification of Annexin A1 (ANXA1), a critical innate immune response protein produced by ciliated airway epithelial cells and macrophages [14]. Because CCSP deficiency in CCSP−/− mice is associated with changes in SCGB3A1, SCGB3A2, and ANXA1, it is unclear if the immune phenotype of CCSP−/− mice is due to CCSP deficiency itself or if it is the result of alterations in protein expressions from cells affected by CCSP deficiency.

The present studies were performed using a murine model of BPD in which neonatal hyperoxic exposure results in arrested alveolarization that mimics the lung pathology seen in human infants with BPD [16–20]. Our data indicated that neonatal hyperoxic exposure followed by room air (RA) recovery caused persistent CCSP deficiency. To determine if proteins previously demonstrated to be altered in CCSP−/− mice were similarly affected by hyperoxia-induced CCSP deficiency, we tested the hypothesis that neonatal hyperoxic exposure persistently alters lung SCGB3A1, SCGB3A2, and ANXA1 expressions in our model. Our data revealed significant decreases in lung SCGB3A1 but not SCGB3A2 protein levels and persistent ANXA1 modifications in RA-recovered mice exposed to hyperoxia during the neonatal period that were associated with evidence of altered immune homeostasis.

## 2. Methods

### 2.1. Animal Studies

Animal protocols were approved by the Institutional Animal Care and Use Committee at the Research Institute at Nationwide Children's Hospital. C3H/HeN mice were bred and pregnant dams were dated as to time of delivery. For analyses of embryonic lung tissues, pregnant dams were sacrificed by cervical dislocation at E19, lungs from the pups were removed, snap frozen in liquid N_2_, and stored at −80°C. For postnatal analyses, at least two dams were required to deliver within 12 hours. Once born, the pups were randomly and equally distributed between the two dams. Half of the dams with litters were placed in a Plexiglas chamber into which 85% O_2_ was delivered at 10 L/min for a maximum of 14 d and the other half remained in room air. The nursing dams were rotated daily between 85% O_2_ and room air to prevent oxygen toxicity in the dams. Pups were sacrificed at 0, 1, 3, 7, or 14 d of life. A subset of mice were removed from hyperoxia at 14 d and raised in RA until 28 d and sacrificed. At the indicated time point, mice were euthanized by a single intraperitoneal injection of 200 mg/kg of sodium pentobarbital. Lung tissues were removed, snap frozen in liquid N_2_, and stored at −80°C. Right lungs from animals sacrificed at 28 d were inflation fixed with formalin at 20 cm H_2_O, paraffin-embedded, and sectioned for immunohistochemical analyses.

### 2.2. Western Blot Analyses

Frozen murine lung tissues were homogenized in lysis buffer as previously described [18], and protein concentrations were determined using a Bio-Rad Protein Assay kit. Proteins (30 *μ*g) were separated on either 4–12% or 12% bis-tris Gels (Invitrogen) and electrophoretically transferred to nitrocellulose membranes (iBlot, Invitrogen). Following blocking with 10% dry milk in tris-buffered saline with Tween 20 (TBS-T), membranes were probed for CCSP or ANXA1 using polyclonal rabbit anti-human CCSP (1 : 5000 in TBS-T; Seven Hills Bioreagents, Cincinnati, OH) or rabbit anti-human ANXA1 (1 : 4000 in TBS-T; Invitrogen) primary antibodies which cross-react with murine CCSP and ANXA1, respectively. Peroxidase-conjugated goat anti-rabbit IgG secondary antibodies (1 : 12,000 in TBS-T; BioRad) were then used. SCGB3A1 and SCGB3A2 were detected using affinity-purified goat anti-mouse/rat SCGB3A1 (1 : 1000 in TBS-T with 5% fish skin gelatin; R&D Systems number AF2954) or goat anti-mouse SCGB3A2 (1 : 500 in TBS-T with 5% fish skin gelatin; R&D Systems number AF3465) primary antibodies, respectively, followed by peroxidase-conjugated donkey anti-goat IgG secondary antibodies (1 : 30,000 in TBS-T with 5% fish skin gelatin; Jackson Immuno Research Laboratories). To control protein loading, membranes were stripped and reprobed with mouse anti-human *β*-actin antibodies (1 : 20000 in TBS-T; Abcam) followed by peroxidase-conjugated goat anti-mouse IgG secondary antibody (1 : 12,000 in TBS-T; Bio-Rad). The membranes were developed using enhanced chemiluminescence (GE Healthcare), and the resulting bands were quantitated using TotalLab software (TotalLab, Ltd). The band density of the protein of interest for each sample was normalized to the density of *β*-actin protein. Exposure time remained uniform for each individual antibody, and all data were reported as ratios of density of the band of interest to density of *β*-actin.

### 2.3. Quantitative Real-Time PCR

The mRNA expression levels of CCSP, SGCL3A1, and SGCL3A2 were measured by quantitative real-time PCR. Briefly, total RNA was isolated from frozen lung tissue using an RNeasy Mini kit (Qiagen, Valencia, CA, USA). cDNA was synthesized using a Maxima First Strand cDNA Synthesis Kit for RT-qPCR (Thermo Scientific Fermentas, K1641, Glen Burnie, MD, USA). Quantitative real-time PCR was performed using Maxima SYBR Green/ROX qPCR Master Mix (Thermo Scientific Fermentas, K0222, Glen Burnie, MD, USA) and the Mastercycler epgradient *Realplex* Real-Time PCR Detection System (Eppendorf, Hamburg, Germany).

### 2.4. Morphometric Analysis

Lungs were inflation fixed with 10% formalin at a pressure of 25 cm H_2_O for 15 min. Fixed tissues were paraffin embedded, sectioned at 5 *μ*m and stained with hematoxylin and eosin (H&E). Five nonoverlapping photomicrographs in different sections were captured at 100x magnification. Images were analyzed using research based digital image analysis software (Image-Pro Plus 6.3; Media Cybernetics, Silver Spring, MD) and a custom macro written for automated alveolar morphometry. 

### 2.5. Two-Dimensional Gel Electrophoresis

Lung homogenates (150 *μ*g) were separated using a ZOOM IPGRunner two-dimensional electrophoresis system (Invitrogen). Briefly, isoelectric focusing (IEF) strips (pH range 3–10) were rehydrated in sample rehydration buffer (8 M urea, 2% Chaps, 50 mM DTT, 0.2% Bio-Lyte, and bromophenol blue) at a total volume of 170 *μ*L. The strips were allowed to rehydrate overnight and were focused using a gradient voltage protocol (175 V for 15 min, 175–2000 V ramp for 45 min, and 2000 V for 30 min). After focusing, the strips were washed two times for 15 min in 5 mL of 1X NuPAGE sample buffer (Invitrogen), loaded onto the second-dimension gel, and separated. Following separation, the proteins were transferred to a nitrocellulose membrane and the membranes were probed for ANXA1 as described above.

### 2.6. Macrophage Immunohistochemistry and Counts

Lung sections obtained from 28-day-old newborn mice were stained with rat anti-mouse Mac3 monoclonal antibody (1 : 500; BD Pharmingen number 550292) followed by rabbit anti-rat (mouse adsorbed) secondary antibody (1 : 200; Vector number BA-4001). The slides were counterstained with hematoxylin. Macrophages were counted in 5 fields for each slide (*n* = 4 mice per group) and averaged.

### 2.7. CCSP Immunohistochemistry

Lung tissue sections were stained with anti-CCSP antibody (1 : 100, Seven Hills Bioreagents) followed by goat anti-rabbit IgG secondary antibodies (1 : 1000; BioRad). The slides were counter stained with hematoxylin.

### 2.8. Statistical Analyses

Data are expressed as mean ± SEM, they were obtained from at least 2 individual experiments except where noted and were tested for homogeneity of variances, by the Shipiro-Wilk test. Data containing 2 groups were analyzed by Student's *t* test for normally distributed data and by Mann-Whitney *U* test where indicated. For multiple comparisons, data were analyzed by two-way ANOVA followed by one-way ANOVA with Tukey's test *post hoc.* All data were analyzed using GraphPad Prism version 5.01 and significance was accepted at *P* < 0.05.

## 3. Results

### 3.1. Newborn Hyperoxic Exposure Causes Persistent Deficits in Lung CCSP Protein Expression

**Figure 1:**
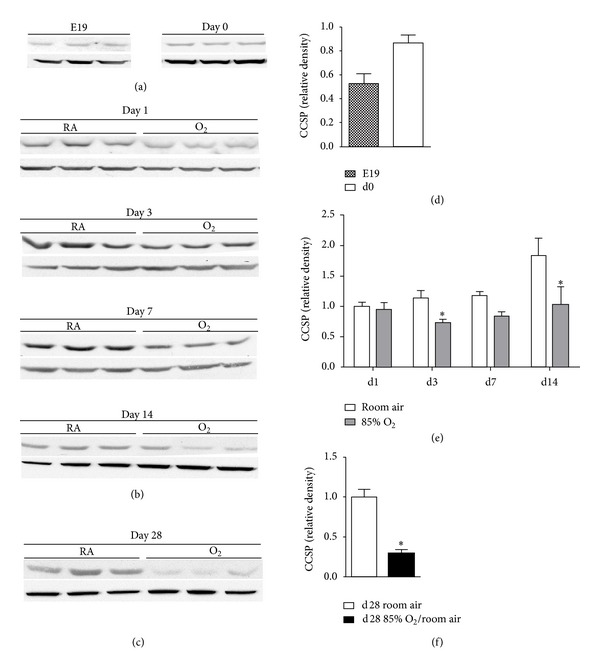
Lung CCSP protein levels. Western blot analyses were performed on lung homogenates prepared from ((a) and (d)) E19 and d0; ((b) and (e)) 1 d, 3 d, 7 d, and 14 d; and ((c) and (f)) 28 d animals as described in Section 2. Animals were exposed to room air or 85% O_2_ as indicated. All data were normalized to d1 room air. Data (mean ± SEM, *n* = 6−9) were analyzed by Mann-Whitney *U* test or two-way ANOVA followed by Tukey's multiple comparison test *post hoc.* Analyses indicated an independent effect of exposure on CCSP levels. (*P < 0.05 versus d1 RA).

Hyperoxia-induced developmental deficits in lung growth were documented by decreased alveolarization (measured as alveolar number per high power field and alveolar area) at both d14 and d28 (see Figure 1 Supplementary Material available at http://dx.doi.org/10.1155/2013/408485). Western blot analyses were performed using lung homogenates from E19, d0, d1, d3, d7, d14, and d28 C3H/HeN mice to determine the developmental ontogeny of lung CCSP expression and the effects of neonatal hyperoxic exposure. Lung CCSP protein levels were 76% greater at d0 following birth into RA than at E19 (Figures 1(a) and 1(d)). The most pronounced developmental increase in lung CCSP protein levels occurred between 7 and 14 d. In animals exposed to RA for 14 d, lung CCSP protein levels were 83% greater than in 1 d newborn pups (Figures 1(b) and 1(e)). Two-way ANOVA indicated an independent effect of hyperoxic exposure on lung CCSP protein contents (Figure 1(e)) in newborn pups continuously exposed to 85% O_2_ from birth through 14 d. At day of life 3, CCSP protein expression was 36% lower in the lungs of hyperoxia-exposed pups than in corresponding RA-exposed controls with a comparable difference at 7 d. The effects of hyperoxia were most pronounced at 14 d with lung CCSP protein levels that were 43% lower in hyperoxia-exposed newborn mice than in RA-exposed controls.

To determine if the effects of neonatal hyperoxic exposure on CCSP protein expression were persistent, we performed western blot analyses for CCSP in lung homogenates from 28 d mice exposed to 85% O_2_ from birth for 14 d followed by RA recovery for 14 d. Lung CCSP protein levels were not different between 14 d and 28 d in control animals. Despite recovery in RA, CCSP protein levels were 71% less in the lungs of 28 d mice exposed to hyperoxia for the first 14 d of life than in 28 d RA control mice (Figures 1(c) and 1(f)).

To determine whether the decreases in CCSP protein levels were due to decreases in production or stability of CCSP protein or as a result of decreases in the numbers of viable Clara cells within the airways; lung tissue sections were immune-stained with CCSP antibodies. Although quantification was not possible due to the heterogeneity of the photomicrographs, there were no obvious differences in Clara cell numbers in the airways of room air or 85% O_2_ exposed mice at 14 d or 28 d (Supplemental Figure 2). 

### 3.2. SCGB3A2 Protein Levels Are Increased by Newborn Hyperoxic Exposure but Normalize with Room-Air Recovery

**Figure 2:**
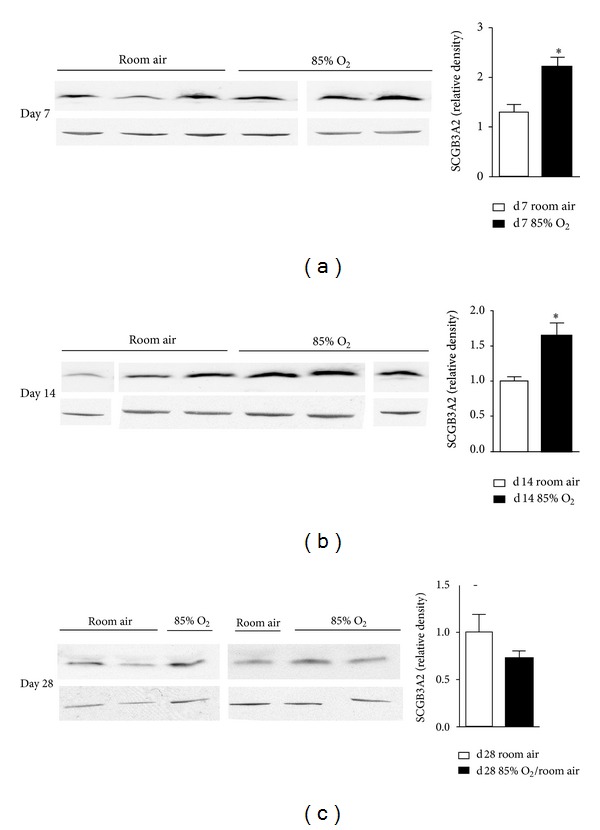
Lung SCGB3A2 protein levels. Western blot analyses were performed on lung homogenates prepared from (a) 7 d, (b) 14 d, and (c) 28 d animals exposed to room air or 85% O_2_ as indicated. All days were normalized to d14 room air. Data (mean ± SEM, *n* = 3–6) were analyzed by Student's *t* test or Mann-Whitney *U* test. (*P < 0.05 versus same day room air).

We determined the effect of hyperoxic exposure on SCGB3A2 protein expression through western blot analyses of lung homogenates from newborn C3H/HeN mice maintained in room air or 85% O_2_ for 7 or 14 d. By 14 d, lung SCGB3A2 protein levels were 36% higher in the lungs of hyperoxia-exposed pups than in 14 d RA controls (Figure 2(b)). In contrast with CCSP, hyperoxia-induced alterations in SCGB3A2 protein levels did not persist following recovery in RA. Our data indicated that 28 d mice recovered in RA for 14 d following exposure to 85% O_2_ for 14 d had levels of lung SCGB3A2 that were not significantly different than in 28 d RA-exposed control mice (Figure 2(c)).

### 3.3. SCGB3A1 Protein Levels Are Persistently Decreased by Newborn Hyperoxic Exposure

**Figure 3:**
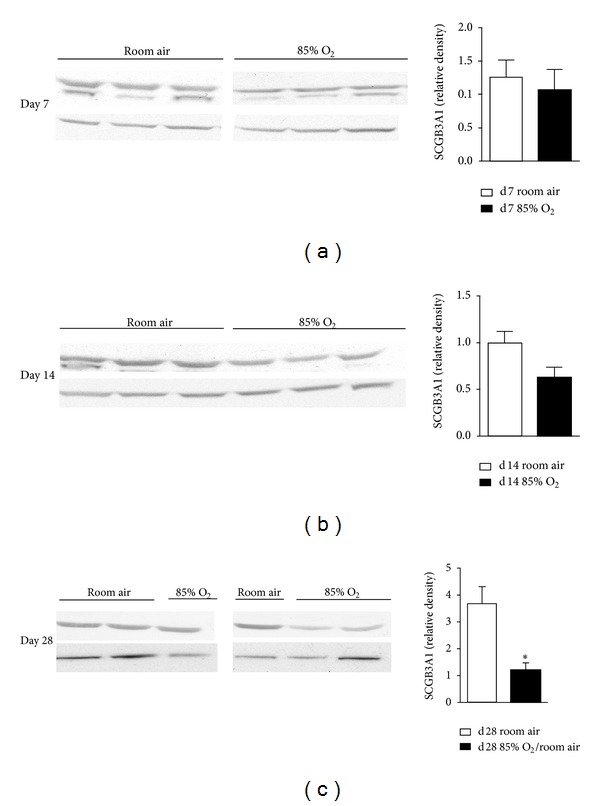
Lung SCGB3A1 protein levels. Western blot analyses were performed on lung homogenates prepared from (a) 7 d, (b) 14 d, and (c) 28 d animals exposed to room air or 85% O_2_ as indicated. All days were normalized to d14 room air. Data (mean ± SEM, *n* = 3–6) were analyzed by Student's *t* test. (*P < 0.05 versus same day room air).

To test the hypothesis that hyperoxic exposure alters SCGB3A1 protein levels in the lungs of hyperoxia-exposed newborn mice, we performed western blot analyses on lung homogenates from pups exposed to RA or 85% O_2_ from birth for 7 or 14 d. SCGB3A1 protein levels were 38% lower in the lungs of 14 d hyperoxia-exposed newborn mice than in the lungs of similarly aged RA controls (Figure 3(b)). Similar to CCSP, neonatal hyperoxic exposure for 14 d caused a persistent deficit in SCGB3A1 protein levels despite 14 d RA recovery. SCGB3A1 protein levels were 67% lower in the lungs of 28 d mice recovered in RA for 14 d following 14 d of hyperoxic exposure than in 28 d RA-raised controls (Figure 3(c)). qRT-PCR analysis indicated that hyperoxia did not induce changes in expression of CCSP, SCGL3A1, or SCGL3A2 mRNA.

**Figure 4:**
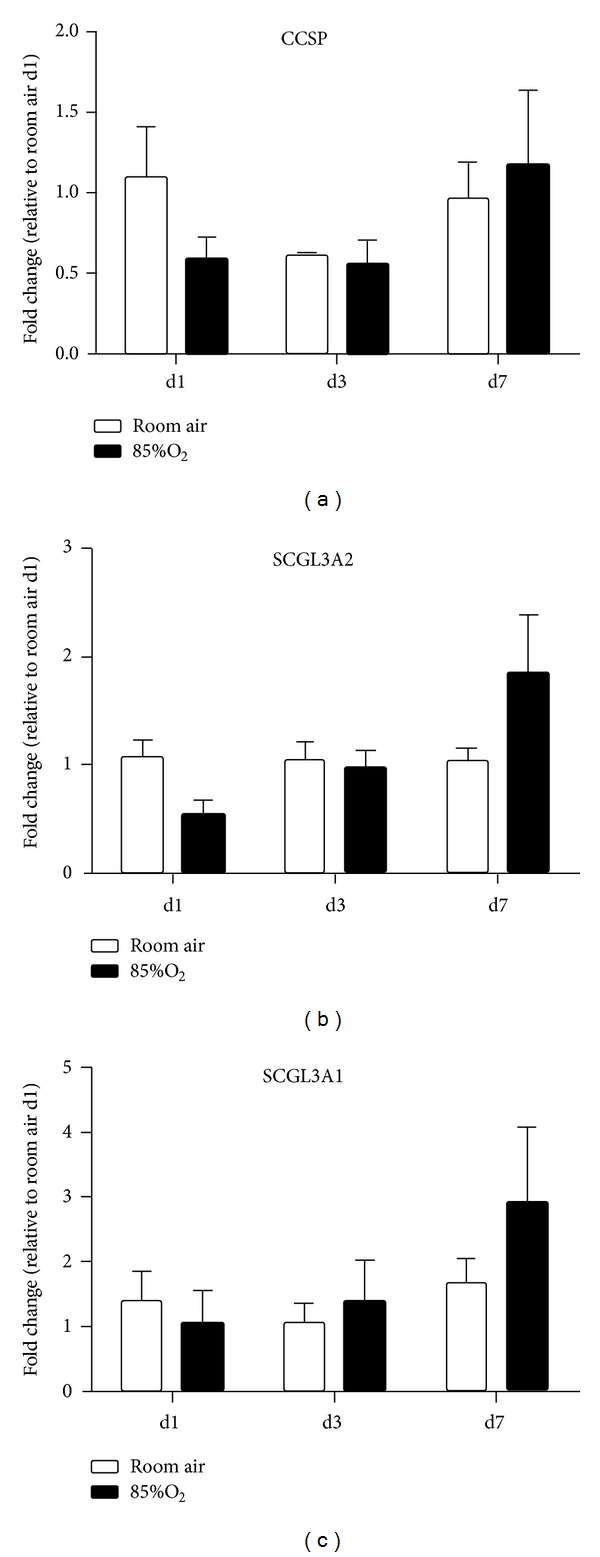
CCSP, SCGB3A2, and SCGB3A1 mRNA levels. mRNA was isolated from tissues obtained from (a) 7 d, (b) 14 d, and (c) 28 d animals exposed to room air or 85% O_2_ as indicated. CCSP, SCGB3A2, and SCGB3A1 mRNA levels were determined by qRT-PCR. Data (mean ± SEM, *n* = 3–6) was normalized to *β*-actin and represent fold change from d1 room air. Data were analyzed by two-way ANOVA followed by Tukey's multiple comparison test *post hoc.* (*P* < 0.05).

To determine if hyperoxia-induced alterations in CCSP, SCGL3A1, or SCGL3A2 protein levels were correlated with altered transcription, and mRNA expression was measured. No effects of hyperoxia or day of life were observed and no individual differences were detected (Figure 4).

### 3.4. Neonatal Hyperoxic Exposure Causes Appearance of Acidic ANXA1 Isoforms

**Figure 5:**
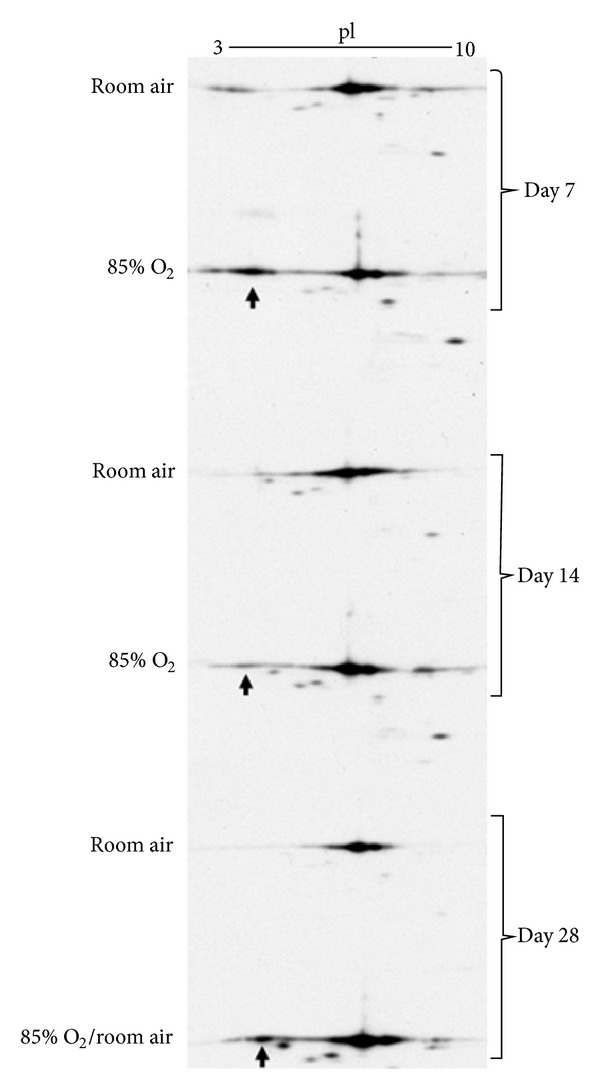
Posttranslational modification of ANXA1. Two-dimensional western blot analyses were performed on lung homogenates from 7 d, 14 d, and 28 d animals exposed to room air or 85% O_2_ as indicated above. The acidic form of ANXA1 is indicated by arrows.

We hypothesized that hyperoxia-induced deficiencies in CCSP protein levels are associated with modifications of ANXA1. Thus, we performed two-dimensional western blot analyses on lung homogenates from 7 d and 14 d newborn mice exposed to RA or 85% O_2_ and 28 d animals exposed to RA for 28 d or 85% O_2_ for 14 d followed by 14 d recovery in RA. Our findings revealed the presence of acidic isoforms of ANXA1 in the lungs of hyperoxia-exposed newborn animals that were less prevalent in the lungs of RA controls at 7 d and 14 d (Figure 5). Furthermore, these ANXA1 modifications were still present at 28 d in the hyperoxia-exposed animal despite RA recovery for 14 d while they were not detected in the 28 d RA control lung. There was no effect of hyperoxic exposure on total lung ANXA1 protein levels (data not shown).

### 3.5. Room Air-Recovered Mice Have Elevated Lung Macrophage Counts

**Figure 6:**
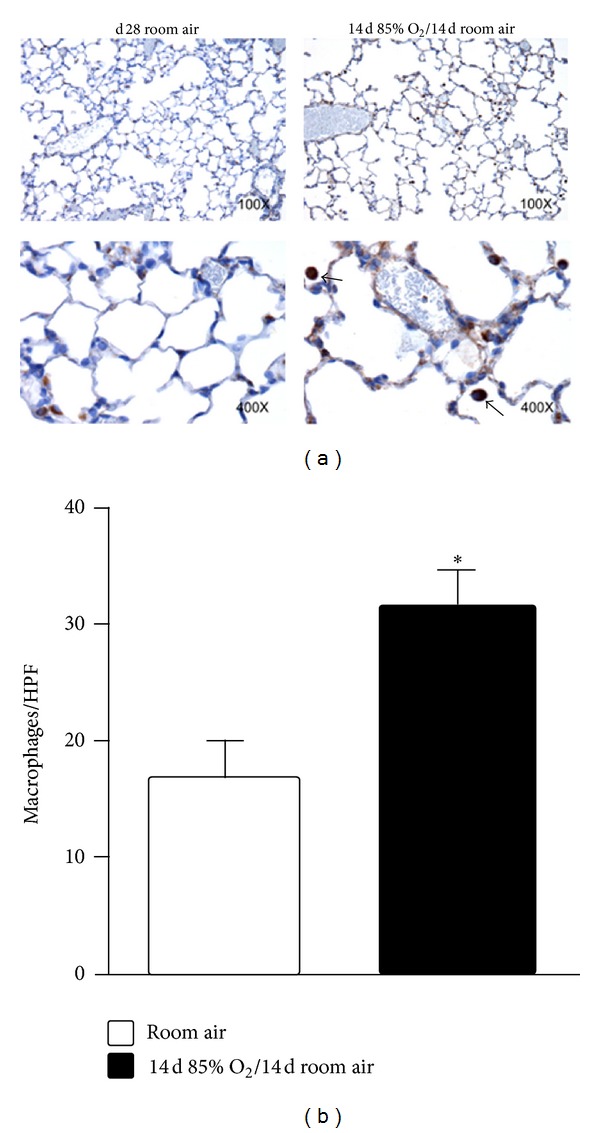
Lung macrophage contents in 28 d mice. Animals were exposed to room air or 85% O_2_ followed by room air as indicated. (a) Representative photomicrographs (100x and 400x). (b) Inflation-fixed sections were stained with anti-Mac3 antibodies and macrophages (indicated by arrowheads) were counted in 5 fields per slide (*n* = 4 mice per group), averaged, and expressed per high-power field (HPF). Data were analyzed by Student's *t* test. (*P < 0.05 versus RA).

To determine the effect of neonatal hyperoxic exposure on macrophage numbers, immunohistochemical analyses for Mac-3, an antigen expressed on the surface of mouse mononuclear phagocytes, were performed on lung sections from 28 d mice exposed to either RA for 28 d or 14 d of 85% O_2_ followed by 14 d RA recovery (Figure 6(a)). Quantitative analyses indicated that 28 d mice exposed to hyperoxia during the neonatal period had 88% more macrophages in the lung than did 28 d RA controls (Figure 6(b)).

## 4. Discussion

We have previously shown that neonatal hyperoxic exposure causes an arrest in alveolar development [17, 18, 20]. The present studies confirm that a relatively short-term exposure to hyperoxia followed by RA recovery that induces persistent alterations in lung structure also alters immune homeostasis which is characterized by: (1) decreased CCSP protein expression; (2) decreased expression of SCGB3A1 protein; (3) increased expression of acidic forms of ANXA1; and (4) increased lung macrophage numbers. We speculate that the combined effects of altered CCSP, SCGB3A1, and ANXA1 proteins reflect changes in epithelial cell biology that are likely to have significant effects on lung innate immune responses and long-term lung health.

Our finding of persistent decreases in CCSP expression following neonatal hyperoxic exposure confirms a previous report by Yee et al. [21] who reported a concentration-dependent inverse relationship between hyperoxia exposure in the neonatal period and CCSP expression in adulthood. The present data, when combined with data from Yee et al., indicate that different O_2_ concentrations (85% versus 40 to 100%) for different durations (14 days versus 4 days) in different strains of mice (C3H/HeN versus C57BL/6J) have similar persistent effects on lung CCSP expression. Collectively, these data suggest that persistent decreases in CCSP expression are likely to be a general airway response to hyperoxia. These changes are likely to have significant biological effects.

CCSP−/− mice express no immunoreactive CCSP protein and exhibit enhanced lung inflammation and injury in response to hyperoxia [9], radiation, and infection with adenovirus [13] or RSV [11]. Furthermore, C57BL/6J adult mice exposed to hyperoxia as neonates and recovered in RA exhibit exaggerated pulmonary immune responses following influenza virus infection [22]. Our finding of elevated macrophage numbers in the lungs of hyperoxia-exposed mice following RA recovery suggests that baseline immune status is altered by neonatal hyperoxic exposure despite RA recovery. Decreased CCSP protein expression did not correlate with decreased mRNA levels. We speculate that the differences in CCSP expression are likely mediated by posttranslational mechanisms.

SCGB3A2 and SCGB3A1 gene expression is confined to distinct subsets of conducting airway epithelial cells. SCGB3A2 is a ubiquitously expressed early molecular marker for Clara cells, is proposed to be a sensitive biomarker of Clara cell function and health, and is expressed by Clara cell populations that also predominantly express CCSP [10]. Conversely, Clara cells in the bronchi predominantly express SCGB3A1. Human infants with BPD have low levels of CCSP, SCGB3A1, and SCGB3A2 gene expression in the surface epithelium of the upper and lower airways suggesting that injury and/or remodeling of airway epithelium that express SCGB family proteins is a pathologic feature of BPD [10]. The findings in infants with BPD differ from findings in CCSP−/− mice in which transcripts of SCGB3A1 and SCGB3A2 are increased [12]. We interpret our finding of persistent decreases in CCSP but not SCGB3A2 to suggest that hyperoxic exposure alters protein production by this distinct population of upper and lower airway Clara cells. Furthermore, we speculate that the increase in SCGB3A2 expression during hyperoxic exposure is indicative of ongoing repair processes given that SCGB3A2 is expressed early in Clara cell development [10, 23] and exhibits growth factor properties [24]. The observed alterations in SCGB family protein expression following neonatal hyperoxic exposure likely represent fundamental alterations in Clara cell biology that are likely to contribute to the observed alterations in innate immune responses previously reported in similar murine models of arrested alveolarization.

Lungs from CCSP−/− mice exhibit increased expression of acidic ANXA1 isoforms. The combination of CCSP deficiency and altered ANXA1 function attributable to these modifications has been speculated to contribute to the hyperinflammatory state observed in chronic lung diseases [14], though little is known about the specific impact of these acidic modifications on ANXA1 function. Though lung epithelial cells and macrophages in the lung express ANXA1, the methods used in the present studies do not allow for the determination of cell-type specificity. Furthermore, while ANXA1 can undergo numerous distinct posttranslational modifications which can alter function and are distinguishable by changes in isoelectric point [14], the specific identities of the alterations are beyond the scope of the present studies. Our finding of decreased lung CCSP expression clearly does not represent complete deficiency; however, alterations in ANXA1 expression suggest that complete CCSP deficiency and attenuated CCSP expression following neonatal hyperoxic exposure have similar effects on ANXA1.

## 5. Conclusions

The mechanisms responsible for altered innate immune responses and enhanced susceptibility to lung infection in BPD remain poorly understood. The studies in the present paper support a model in which Clara cells are important mediators of the altered airway epithelial cell immune function in both human and experimental BPD. Infants with BPD have impaired lung innate immune responses and altered mucosal barrier defenses negatively impact lung innate immunity. Clinically relevant interventions designed to protect airway epithelial cell immune function by preserving Clara cell function have the potential to decrease the respiratory morbidities that afflict patients with BPD. While the present studies were not designed to delineate the mechanism(s) by which neonatal hyperoxic exposure alters the abundance of Clara cell subpopulations within the lung and/or protein production by Clara cells, our findings suggest the need for follow-up studies to determine the specific roles of SCGB family proteins and ANXA1 in lung innate immune responses in BPD.

## Supplementary Material

Supplementary Figure 1 contains lung histologic analyses from newborn mice exposed to room air or hyperoxia for 14 days. Separate groups of mice were exposed to room air for 28 days or was exposed to 14 days of hyperoxia followed by exposure to room air for an additional 14 days. Formal morphometric analyses from 28 d mice are also shown.
Supplementary Figure 2 contains immunohistochemical analyses for Clara cell secretory protein (CCSP) in lung sections obtained from newborn mice exposed to room air for 28 days or exposed to hyperoxia for 14 days followed exposure to room air for an additional 14 days.

## Abbreviations

BPD: Bronchopulmonary dysplasia
SCGB: Secretoglobin
CCSP: Clara cell secretory protein
ANXA1: Annexin A1
CCSP−/−: CCSP knockout
RA: Room air
RSV: Respiratory syncytial virus.
