# Supplementary material for: Neonatal Hyperoxic Exposure Persistently Alters Lung Secretoglobins and Annexin A1
Source: Biomed Res Int. 2013 Sep 26;2013:408485. doi: 10.1155/2013/408485 (PMC3804154; doi:10.1155/2013/408485)
Supplement: Supplementary file 1 — Supplementary Figure 1 contains lung histologic analyses from newborn mice exposed to room air or hyperoxia for 14 days. Separate groups of mice were exposed to room air for 28 days or was exposed to 14 days of hyperoxia followed by exposure to room air for an additional 14 days. Formal morphometric analyses from 28 d mice are also shown. Supplementary Figure 2 contains immunohistochemical analyses for Clara cell secretory protein (CCSP) in lung sections obtained from newborn mice exposed to room air for 28 days or exposed to hyperoxia for 14 days followed exposure to room air for an additional 14 days. [file 408485.f1.docx]

Supplemental Figure 1


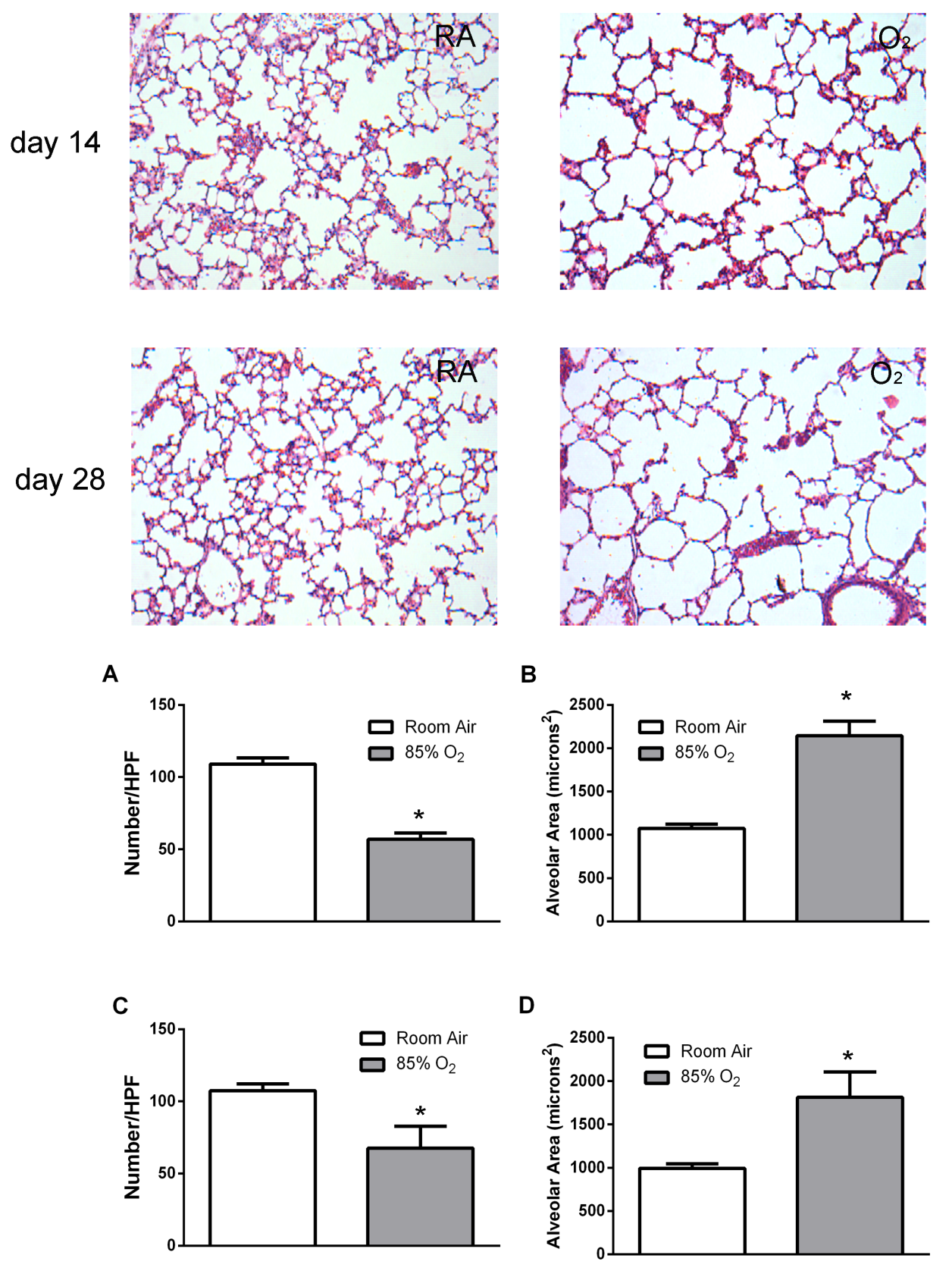


Supplemental Figure 2


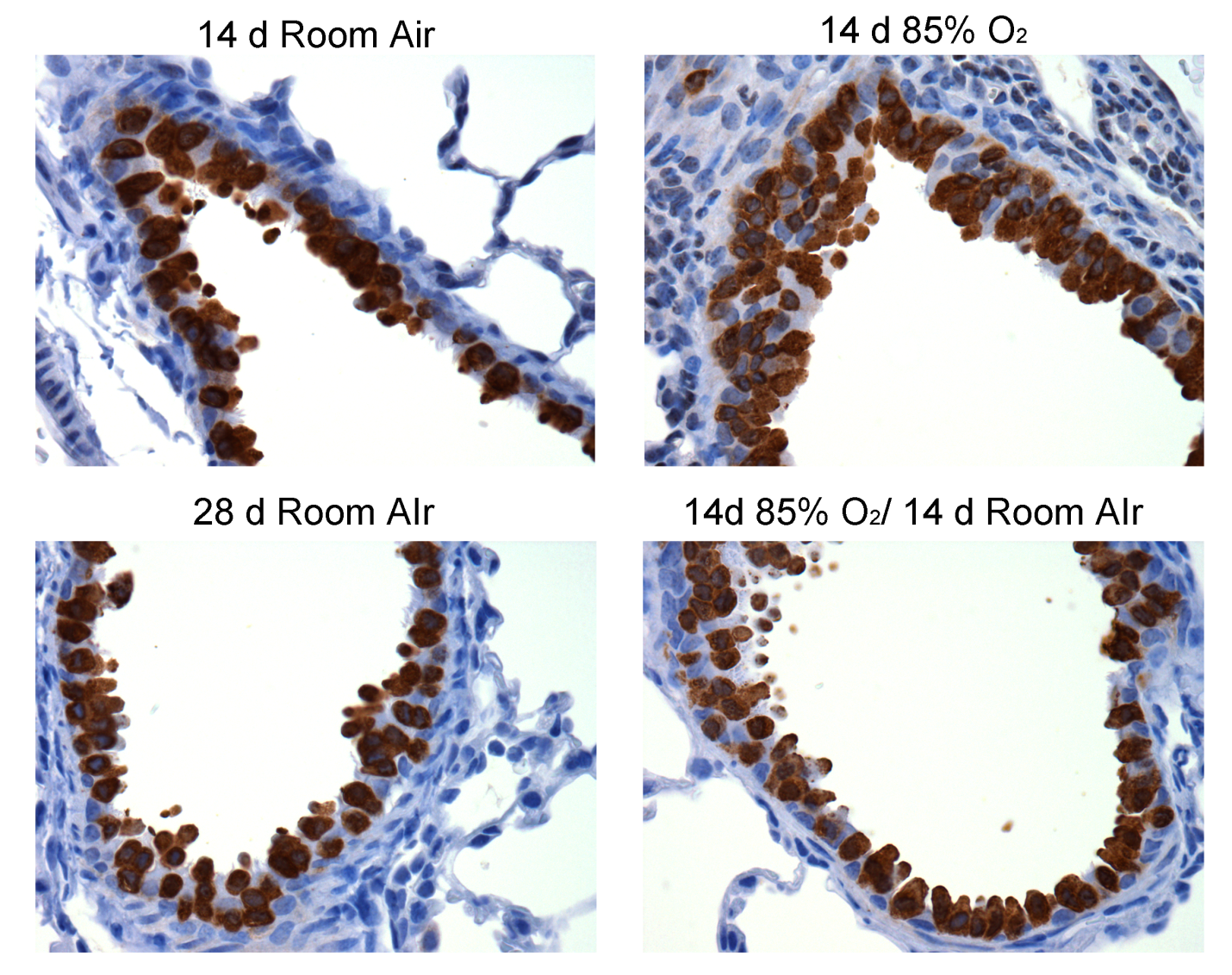


**Supplemental Figure 1.** Fixed lung tissues obtained from d28 mice were paraffin embedded, sectioned at 5 μm and stained with hematoxylin and eosin (H&E). Five non-overlapping photomicrographs were captured at 100x magnification. Images were analyzed using research based digital image analysis software. Data (mean±SEM, n=3-4) were analyzed by Student’s t-test. (*p<0.05 vs RA).

**Supplemental Figure 2.** Fixed lung tissues obtained from d28 mice were paraffin embedded, sectioned at 5 μm and treated with anti-CCSP antibody as described in Methods. Images were obtained at 400x and indicated CCSP containing cells in airways.
